# Longitudinal analysis of the hand microbiome in response to chlorine-based antiseptic use during a military field exercise

**DOI:** 10.1128/spectrum.03771-25

**Published:** 2026-07-14

**Authors:** Susanne Glenna, Einar E. Birkeland, Russell J. S. Orr, Gregor D. Gilfillan, Marianne Dalland, Ole Andreas Økstad, Øyvind A. Voie, Trine B. Rounge

**Affiliations:** 1 Section for Pharmacology and Pharmaceutical Biosciences, Department of Pharmacy, University of Oslo 6305, Oslo, Norway; 2 Norwegian Defense Research Establishment (FFI), Kjeller, Norway; 3 Department of Medical Genetics, Oslo University Hospital and University of Oslo 6305, Oslo, Norway; 4 Cancer Registry of Norway, Norwegian Institute of Public Health 25563, Oslo, Norway; Xi'an Jiaotong-Liverpool University, Suzhou, Jiangsu Province, China; Chengdu Institute of Biology, Chengdu, Sichuan, China

**Keywords:** skin microbiome, microbiota, 16S rRNA sequencing, military personnel, hygiene, infection control, hand sanitizers, hypochlorous acid

## Abstract

Hand hygiene is essential for infection control, yet the impact of frequent antiseptic use on the skin microbiome, which is crucial for skin barrier function and pathogen exclusion, remains underexplored, especially in field conditions. In high-risk military settings, there is a need for safe, multipurpose antiseptics that avoid the drawbacks of alcohol-based options, including skin irritation, flammability, and unsuitability for wound application. We conducted a longitudinal study during the Norwegian-led military exercise Cold Response 2022 to assess the effects of repeated use of stabilized hypochlorous acid, a non-alcoholic antiseptic, on the hand microbiome of soldiers in a field environment. Participants used either the chlorine-based antiseptic (*n* = 20) or standard hygiene practices, mainly alcohol-based sanitizers (*n* = 19), for 10 days. Skin swabs from hands and untreated forearms were collected at baseline, post-intervention, and three weeks after discontinuation, and analyzed using 16S rRNA sequencing. Overall, our data revealed that field exposure accounted for more variation in bacterial composition than antiseptic use. When comparing alpha and beta diversity trajectories between the two antiseptic groups, there was minimal divergence during active field use, but a significant post-treatment increase in alpha diversity associated with the use of chlorine-based antiseptics. In contrast, untreated forearms showed no such group-level differences. These findings suggest that antiseptic-driven microbiome shifts are most apparent during recovery, with non-alcoholic formulations associated with faster recolonization after treatment. Stabilized hypochlorous acid represents an alternative to standard alcohol-based antiseptics for military personnel.

**IMPORTANCE:**

Military personnel often rely on frequent hand antisepsis in field environments where infection risk is elevated and products may need to be used on both intact and damaged skin. Yet the effects of repeated antiseptics on the skin microbiome are poorly understood. To our knowledge, this is the first longitudinal study comparing hand microbiota trajectories following repeated antiseptic use in a real-world operational setting. Microbiome changes were most evident after treatment cessation than during active use, and the non-alcoholic hypochlorous acid-based antiseptic was associated with greater recovery of bacterial diversity. These findings show the value of longitudinal sampling and may have implications for infection control and antiseptic selection in military and other high-risk settings.

## INTRODUCTION

The skin acts as a protective barrier against environmental threats, with its microbiota - comprising both resident and transient microorganisms - playing a central role in maintaining cutaneous homeostasis and preventing pathogen colonization (1–3). Among all skin sites, the hands are uniquely exposed to external influences, making their microbial communities highly dynamic and sensitive to factors such as hygiene practices (2, 4, 5). Hand hygiene is one of the most effective measures for reducing pathogen transmission (6–9), yet the effects of handwashing and sanitizer use on microbial diversity and resilience across different populations remain poorly understood (10–13). This limits our ability to balance hygiene with microbiome preservation. Most antiseptics, such as chlorhexidine and povidone-iodine, are used in a broad-spectrum, non-selective manner, reducing microbial burden indiscriminately across taxa (14, 15). While effective for infection control, such non-targeted action can also disrupt commensal as well as pathogenic populations and alter community composition, with recovery patterns varying by agent and context (10, 11, 16). As the global use of topical antimicrobial agents continues to rise (17), longitudinal studies are needed to evaluate their effects on a microbial community-wide scale (18, 19).

In military field conditions, where infection and injury risks are elevated, good personal hygiene and effective antimicrobial agents are critical (20–24). Military personnel commonly use alcohol-based products for disinfecting hands and surfaces, which are vital for preventing infections (25, 26). However, concerns remain about their safety, as these disinfectants may cause skin irritation, especially in sensitive or compromised skin, leading to discomfort and reduced compliance (27–29). Ethanol, a common active ingredient, is irritating to wounds and mucosal surfaces, toxic if ingested, and highly flammable (30, 31). Despite these limitations, alcohol-free alternatives have not yet been successfully implemented in the defense sector. Moreover, current field protocols require multiple products for disinfection and wound care, highlighting the need for a multipurpose, alcohol-free antiseptic (25). A promising candidate is a novel combination of two well-known antimicrobial substances: hypochlorous acid (HOCl), a naturally occurring oxidant produced by our immune system (32–34), and acetic acid, which has historically been used in medicine (35–37) and acts as a buffer to stabilize HOCl at its optimal pH (32, 38). Both compounds exhibit broad-spectrum antimicrobial and anti-biofilm activity, supporting their use in treating infected wounds and skin conditions such as eczema (37, 39–44).

Despite the demonstrated *in vitro* antimicrobial activity (32, 39, 45), the influence of stabilized HOCl on the skin microbiome under real-world conditions is unknown. This study aims to explore how field application of stabilized HOCl affects the human hand microbiota by using 16S rRNA amplicon sequencing to assess shifts in bacterial communities in response to hand sanitation. We previously characterized the hand and forearm microbiota of soldiers following a 10-day field exercise with standard hygiene practices, revealing community shifts with enrichment of soil- and water-associated bacteria (46). Building on this, the current study investigates whether HOCl-based antiseptic use during field exercise alters hand bacterial composition and diversity beyond environmental effects. We directly compare microbiome trajectories of the HOCl intervention group to the previously studied control group and evaluate both immediate and sustained effects of repeated antiseptic application under real-world field conditions.

## MATERIALS AND METHODS

### Study design and participants

**Fig 1:**
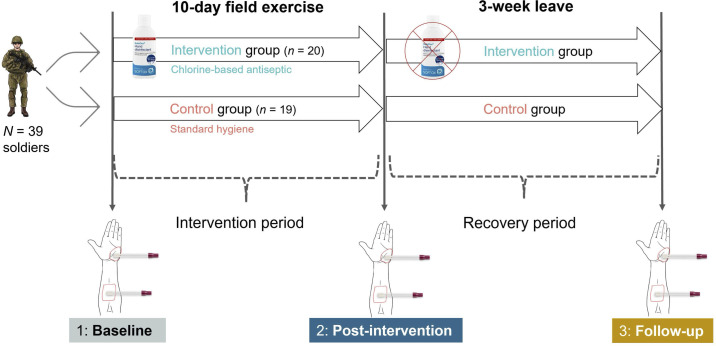
Study design. Soldiers (*n* = 39) participating in a 10-day field exercise were sampled for skin microbiome analysis at three time points: (1) baseline (pre-exercise), (2) post-intervention (immediately after exercise/treatment), and (3) follow-up (3 weeks later). Participants were assigned to an intervention group (blue) using chlorine-based antiseptic or a control group (pink) maintaining standard hygiene during the exercise. Skin swabs from hands (treated) and forearms (untreated) were collected at each time point. Periods between sampling are termed intervention (exercise plus antiseptic use) and recovery (3 weeks post-use).

Norwegian soldiers participating in NATO’s “Cold Response” exercise in Innlandet County of Norway in March 2022 were recruited for this longitudinal study. Participants were assigned to either a chlorine-based antiseptic intervention group or a control group following their regular hygiene practices, based on existing troop affiliations (Fig. 1). Both groups operated in the same geographical area and engaged in similar tasks during the exercise, ensuring comparable environmental exposures. The intervention group received a hypochlorous acid + acetic acid hand disinfectant (concentrations specified below) along with standardized instructions: apply ~3 mL per use, ensure full hand coverage, allow air-drying, and apply before and after meals, as well as after restroom visits over the 10-day field exercise. Each participant was issued two 100 mL flasks labeled with study IDs, weighed before and after the exercise to quantify individual usage. Participants were instructed not to share flasks and to use only the provided product. The control group received no guidance on antiseptic use and was not given access to the chlorine-based product. To avoid interfering with established routines or compromising hygiene, control group participants maintained their typical practices, which were documented. Both groups underwent identical sampling protocols at three time points (Fig. 1): (i) baseline (the day before the exercise), (ii) post-intervention (after the 10-day field exercise with or without antiseptic intervention), and (iii) follow-up (3 weeks after the end of exercise and antiseptic intervention). Two skin sites were sampled: the hands, which received antiseptic treatment, and the forearms, serving as untreated intra-individual controls. Exclusion criteria included self-reported antibiotic use within 6 months before enrollment, observable dermatologic conditions, immunocompromised states, and other self-reported illnesses. Three participants were excluded from the second and third samplings due to COVID-19 illness, and two missed the follow-up for unrelated reasons. Participants were instructed to refrain from showering, handwashing, or using skincare products for 12 h before each sampling session. At each session, participants completed a questionnaire including questions related to their demographics, health, activity, and antiseptic usage.

### Antiseptic intervention product

The active chlorine hand disinfectant (SafeDes, SoftOx Solutions AS, Norway) contains 0.02% hypochlorous acid (HOCl), stabilized in 0.25% acetic acid (HOAc), at a pH of 4.3. It is approved according to EN 1500, EN 13727, and EN 13624.

### Sample collection

Skin samples were collected from the participants at three time points following a standardized protocol previously described (46). Briefly, skin swabs were taken from the hypothenar region of the palms and the volar forearms (Fig. 1) using sterile, pre-wetted, dual-flocked swabs. For each skin site, the left and right sides were pooled into a single sample using the same swab. Negative control swabs were included in each sampling event. All samples were immediately stored at −80°C until further processing.

### DNA extraction and 16S rRNA sequencing

The DNA extraction, library preparation, and sequencing were performed concurrently for both studies of the complete data set, with all samples mixed randomly and processed collectively (46). In brief, total DNA was extracted from skin samples using the NucleoSpin 96 Soil kit (Macherey-Nagel). Each swab pair was subjected to chemical (SL2 lysis buffer) and mechanical (bead-beating) lysis, after which the lysates were combined for DNA binding and purification using a spin column. Amplicon-based sequencing of the extracted DNA was performed using the Illumina protocol (47), targeting the 16S V3-V4 region with 341F/785R primers (48). Libraries were sequenced on an Illumina MiSeq v3 with 300 bp paired-end reads.

### Bioinformatics analyses

Microbial community analyses were performed using the bioinformatics pipeline described in reference 46. Briefly, we performed quality control and trimming of the 16S amplicon reads with MultiQC (v.1.7) (49), Trim Galore (v.0.6.6) (50), and Cutadapt (v.4.2) (51). Samples with sufficient trimmed reads (>5,000) were imported into QIIME 2 (v.2024.2) (52) for taxonomic analysis, where amplicon sequence variants (ASVs) were inferred with DADA2 (53) using truncation settings optimized to retain high-quality overlapping pairs. Taxonomy was assigned with the SILVA 16S rRNA v.138 database (54) using a V3-V4 trained sklearn classifier (55). Mitochondrial-, chloroplast-, and singleton reads were removed prior to import into R for subsequent microbiome analyses using phyloseq (v.1.42.0) (56).

Taxonomic classification accuracy was previously validated using mock communities processed alongside all study samples, as described in reference 46. The Decontam R package was used to identify and remove contaminating ASVs (Fig. S1). Samples were rarefied to 7,000 reads based on rarefaction curve saturation (Fig. S2). Owing to consistently lower input DNA in forearm swabs, a large proportion of these samples did not meet the minimum read-depth and quality thresholds and were thus excluded, as shown in Table 1.

**TABLE 1:** Characteristics of study participants (*n* = 39) and samples in control and intervention groups

Variables	Control (*n* = 19)	Intervention (*n* = 20)
Age^*a*^	23.6 ± 3.1 (20–30)	24.7 ± 4.2 (21–38)
BMI^*a*^	25.2 ± 1.5 (21.6–27.8)	25.2 ± 1.7 (23.0–28.1)
Employment time in the armed forces	≤ 3 years: 70% >3 years: 30%	≤ 3 years: 64% >3 years: 36%
Daily snuff users, *n* Daily smokers, *n*	9 0	8 0
Asthma, allergic rash, pollen or food allergies, *n*	9	4
Living with pets^*b*^, *n*	2	9
Prior regular weekly workouts, *n*		
< 1 day	1	0
1–3 days	1	1
3–5 days	6	9
5–7 days	11	10
Prior regular use, antiseptic type		
Ethanol-based	15	20
Soap and water (only)	4	0
Antiseptic used during military exercise	Ethanol-based: 16 None: 1	Chlorine-based: 19
Antiseptic application times during military exercise	Never: 1 1–5: 14 6–10: 2	Never: 0 1–5: 19 6–10: 0
Fraction of time wearing gloves during military exercise, *n*		
Never	0	0
10%–40%	0	6
40%–80%	11	9
Always	6	4
Total skin samples^*c*^, *n*	81 (102)	82 (110)
Hand samples^*c*^, *n*		
Baseline	19 (19)	19 (20)
Post-intervention	17 (17)	19 (19)
Follow-up	15 (15)	16 (16)
Forearm samples^*c*^, *n*		
Baseline	8 (19)	5 (20)
Post-intervention	14 (17)	16 (19)
Follow-up	8 (15)	7 (16)

^*a*^ Average values with standard deviations are reported, including the range in brackets.

^*b*^ Living with pets. Control group: one with a cat, one with a cat and a dog. Intervention group: seven with dogs, two with cats.

^*c*^ The number of samples after processing of sequence data (including trimming and rarefaction), as well as total collected in parenthesis.

### Statistical analysis

Statistical analyses were done in R (v.4.4.1) using ggplot2 (v.3.5.0) for visualizations (57). In all statistical models, the DNA extraction batch was included to adjust for technical biases, and associations were considered significant at the *P* < 0.05 level. Each skin site (hands and forearms) was analyzed separately.

Alpha (within-sample) diversity was assessed using Shannon and Inverse Simpson indices. Linear mixed models were fitted using the nlme package (v.3.1-165) to test differences in alpha diversity over time, with a random intercept for subject ID to account for repeated measures. Model assumptions were checked by inspecting residuals for normality and heteroscedasticity, and model fit was evaluated using the Akaike Information Criterion (AIC). Competing models were compared using likelihood ratio tests via ANOVA to assess whether increased complexity improved fit. To test for group-by-time interaction, we used the model: alpha diversity ~ group + time_point + group:time_point + batch, random = ~1 | subject. Estimated marginal means for the fitted model were computed using the emmeans R package (v.1.8.9), with custom contrasts to compare the difference-in-differences between the two groups. Multiple comparisons were adjusted with the Tukey method. As the intervention group had more pet owners (Table 1), we added pet ownership as a fixed effect in a sensitivity analysis. To test for a dose-response relationship between antiseptic use and alpha diversity after intervention, we used the following model: post_alpha ~ antiseptic amount + group + baseline_alpha, random = ~1 | subject, including baseline diversity as a covariate to adjust for initial differences.

Beta diversity was evaluated using Bray-Curtis dissimilarities. Associations were tested using PERMANOVA (999 permutations) with the adonis2 function from the vegan package (v.2.6-4), incorporating a group × time point interaction and subject-level stratification to account for repeated measures in the treated skin site (58). For the forearm site, which had sparse repeated measures, subject ID was included as a covariate in the model to adjust for individual-level variation. Group dispersions were assessed with PERMDISP, using vegan’s betadisper (type = “centroid”) followed by permutest (999 permutations) for pairwise comparisons. When PERMDISP revealed unequal variance between groups, ANOSIM was used as a complementary test. Intra-individual Bray-Curtis changes between groups were compared using the Wilcoxon rank-sum test. A linear regression model was used to determine whether antiseptic volume (scaled) predicted changes in beta diversity within the intervention group.

Differential abundance analysis of raw ASV counts was performed with DESeq2 (59) as described earlier (46), only now with the following design formula: design = ~ batch + group + time_point + group:time_point, where the group:time_point interaction identified taxa with differential trajectories in the intervention versus control groups through pairwise contrasts between time points. ASVs with an FDR-adjusted (Benjamini-Hochberg) *P* value <0.05 and absolute log_2_ fold change ≥1 in the interaction term were considered statistically significant. The log_2_ fold change reflects how the change in abundance over time differs between groups; positive values indicate that taxa increased more (or decreased less) in the intervention group compared to the control group, whereas negative values indicate that taxa increased more (or decreased more strongly) in the control group compared to the intervention group. ComplexHeatmap (v.2.14.0) (60) was used to generate a heatmap of differentially abundant taxa.

## RESULTS

### Study cohort and sequencing overview

All study participants (*n* = 39) were healthy Norwegian male professional soldiers, aged 20 to 38 (median age 23), and the two groups (control, *n* = 19; intervention, *n* = 20) had comparable baseline characteristics (Table 1).

Participants in the intervention group confirmed exclusive use of the chlorine-based antiseptic over the 10-day field exercise. Flask weights showed a median use of 58 mL (range: 14 mL–128 mL), corresponding to an estimated 19 total applications (based on 3 mL per use), or approximately two applications (range: 1–5) per day. At post-intervention, seven of 19 reported that their hands felt better after using the novel product, while the rest experienced no change in comfort compared to regular practice. Participants in the control group reported using exclusively 85% ethanol-based disinfection products during the exercise, with the majority (>80%) reporting one to five applications per day (Table 1). Five out of 16 reported having dry hands after using ethanol-based antiseptic.

From a total of 212 skin swab samples collected and 16S sequenced, 163 samples (control, 81; intervention, 82) remained after all quality-filtering steps and rarefaction (Table 1). In total, these retained samples yielded 5,295,318 high-quality paired-end reads, with a median of 29,216 reads per sample (range: 9,809–74,416). Rarefaction curves indicated that a sequencing depth of 7,000 reads was sufficient to capture microbial diversity (Fig. S2). Attrition was particularly pronounced for forearm samples, with 38/106 excluded during read processing (Table 1), thereby reducing statistical power for analysis at this untreated control site. The final data set comprised 8,536 ASVs, with a median of 329 ASVs per sample (hands: median 394, range 87–932; forearms: median 146, range 44–509).

### Alpha diversity

**Fig 2:**
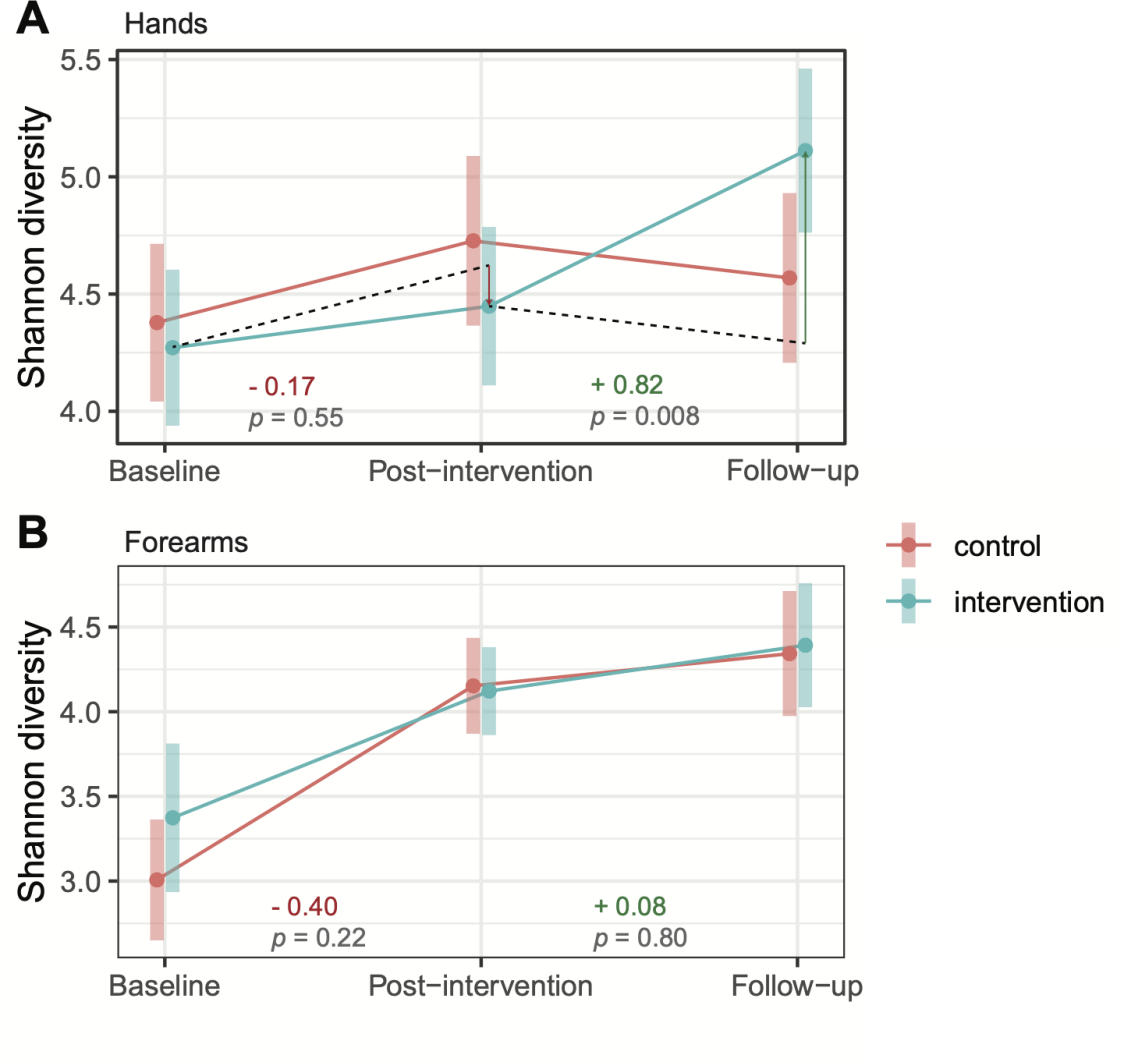
Alpha diversity for group × time point. Interaction plot of Shannon diversity (estimated marginal means) across groups (pink = control, blue = intervention) and time points (baseline, post-intervention: after 10 days of antiseptic use, and follow-up 3 weeks after cessation) in (**A**) hands and (**B**) forearms (untreated control site). The interaction estimate for each time period is annotated along with the *P* value. Error bars indicate 95% confidence intervals around the means. Dotted lines in panel **A** demonstrate the counterfactual (where the intervention group would be without the intervention), and arrows are added for visualization of the treatment effect.

We investigated temporal changes in alpha diversity on the hands between the chlorine-based intervention group and the control group. A linear mixed model revealed a significant group × time-point interaction (ANOVA, F = 4.05, *P =* 0.022), indicating differing trajectories of Shannon diversity (Fig. 2). Corresponding results were observed with the Inverse Simpson index (Fig. S3). Initially, we compared baseline to post-intervention changes to assess the immediate effect of antiseptic use. No significant reduction in diversity was found in the intervention group relative to the controls immediately after the 10-day use (Shannon estimate: −0.17, *P =* 0.55; Inverse Simpson estimate: −10.2, *P =* 0.45; Fig. 2A), and antiseptic dosage showed no significant association with post-intervention diversity (effect size Shannon = 0.004, *P =* 0.42). However, during the recovery phase (from day 10 to the 3 week follow-up), the intervention group exhibited a significant increase in diversity compared to the control group (Shannon estimate: +0.82, *P =* 0.0083; Inverse Simpson estimate: +45.8, *P =* 0.0018; Fig. 2A and Fig. S3, respectively). The impact of pet ownership on the model was evaluated using a likelihood ratio test comparing the original model with a pet-adjusted model, showing that inclusion of pet ownership as a fixed effect did not significantly improve model fit (ΔAIC = +2, L.ratio = 0.026, *P* = 0.87). Moreover, the overall group × time interaction remained significant in the adjusted model (F = 4.058, *P* = 0.022), and the coefficient for pet ownership was negligible and non-significant (F = 0.0021, *P* = 0.9636).

Forearm samples served as an internal control site, as the antiseptic was applied only to the hands. There was no statistically significant interaction between group and time on alpha diversity at this site (F = 0.80, *P =* 0.46), with no difference detected in the change between groups in either time period (*P =* 0.22, *P =* 0.80; Fig. 2B). Regardless of treatment, both groups exhibited increased forearm diversity during the exercise (*P* < 0.001, Fig. 2B and Fig. S4).

### Beta diversity

**Fig 3:**
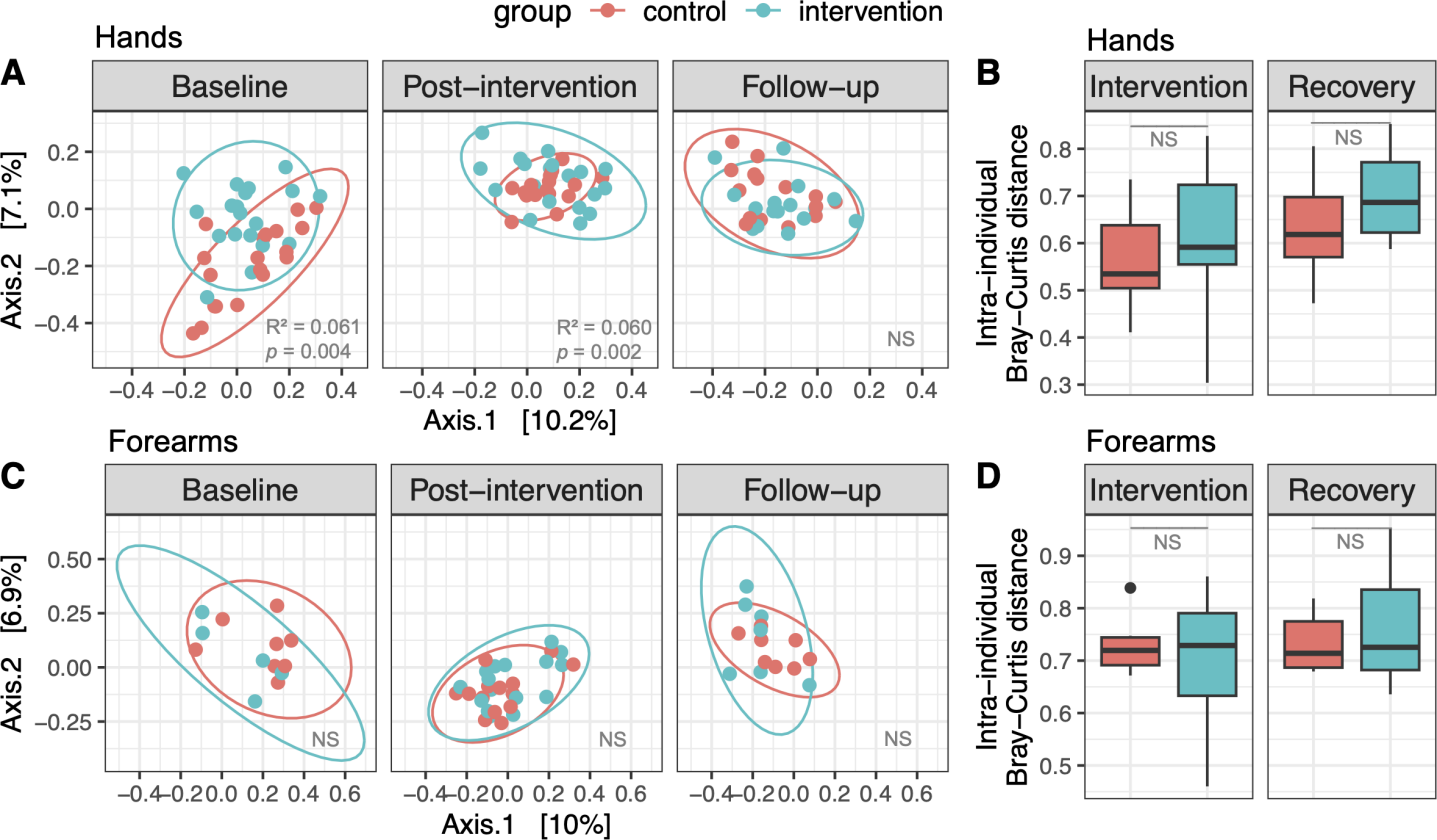
Beta diversity assessed by Bray-Curtis dissimilarity**.** (**A and B**) Hand samples; (**C and D**) forearm samples. (**A and C**) Principal Coordinates Analysis (PCoA) plots showing group separation (95% CI); points represent samples colored by group (pink = control, blue = intervention). Axes indicate principal components with explained variance (%). Proximity of points indicates high similarity in the microbial community. PERMANOVA: *R*² is the proportion of variance explained by group differences, with significant differences reported for *P* < 0.05. NS: non-significant. (**B and D**) Box plots of intra-individual distances over the two time periods (intervention: baseline to post-intervention; recovery: post-intervention to follow-up) for each group. Wilcoxon rank-sum test used to compare groups, NS, non-significant.

To assess whether hand disinfection altered beta diversity beyond the military exercise-induced changes observed in the control group (46), we fitted a PERMANOVA model including a group × time-point interaction. The full model revealed a significant interaction effect across all time points (*R*^2^ = 0.026, *P* = 0.003), as well as an effect of time point (*R*^2^ = 0.092, *P* = 0.002), suggesting that temporal trajectories differed between groups, but that the treatment effect was smaller than that of field exercise and recovery (Fig. 3A). When restricting analysis to consecutive two-time point subsets, the interaction effect remained significant during the intervention period (*R*^2^ = 0.023, *P* = 0.004), but not during the recovery period (*R*^2^ = 0.014, *P* = 0.17). The untreated forearm site showed no significant group differences over time (*R*^2^ = 0.028, *P* = 0.44), supporting the hand-specificity of the intervention (Fig. 3C and 3D). Despite these findings, interpretation of a true treatment effect was complicated by a significant difference between the two groups at baseline (*R*² = 0.061, *P* = 0.004; Fig. 3A). Groups were also different at post-intervention (*P* = 0.002), but with a similar amount of variation explained (*R*² = 0.060). At follow-up, the groups showed no significant differences (*R*² = 0.038, *P* = 0.24). Exploring temporal dynamics within groups, the intervention group exhibited lower variance explained by time point (*R*^2^ = 0.10) compared to controls (*R*^2^ = 0.16), suggesting smaller community shifts at the group level. At the individual level, however, there was a tendency for greater changes (intra-individual distances) in the intervention group (Fig. 3B), although this was not statistically significant, neither during the intervention (*P* = 0.3) nor during recovery (*P* = 0.1). Moreover, the amount of antiseptic used by the intervention group was not a significant predictor of beta diversity changes (estimate = 0.023, SE = 0.033, *P =* 0.49).

To assess if group differences in beta diversity were due to changes in variability, we analyzed beta dispersion using PERMDISP. The analysis confirmed overall heterogeneity differences between groups (ANOVA: F = 7.9, *P* < 0.001), with pairwise tests identifying a significant dispersion difference only at post-intervention (permuted *P* = 0.010; Fig. S5A). Accounting for this, ANOSIM confirmed significant group separation at post-intervention (*R* = 0.12, *P =* 0.003) beyond dispersion effects. Within groups, only the control group exhibited changes in dispersion across time points (ANOVA, F = 13, *P* < 0.001), whereas the intervention group remained comparatively stable (ANOVA, F = 2.8, *P* = 0.07). Both groups appeared more homogeneous at post-intervention (Fig. S5A), although reductions in distances to centroids were not significant (*P* > 0.05). At follow-up, both groups showed similarly elevated dispersion (Fig. S5A). Comparable patterns of beta dispersion were seen at the forearm site (Fig. S5B).

### Differential abundance

**Fig 4:**
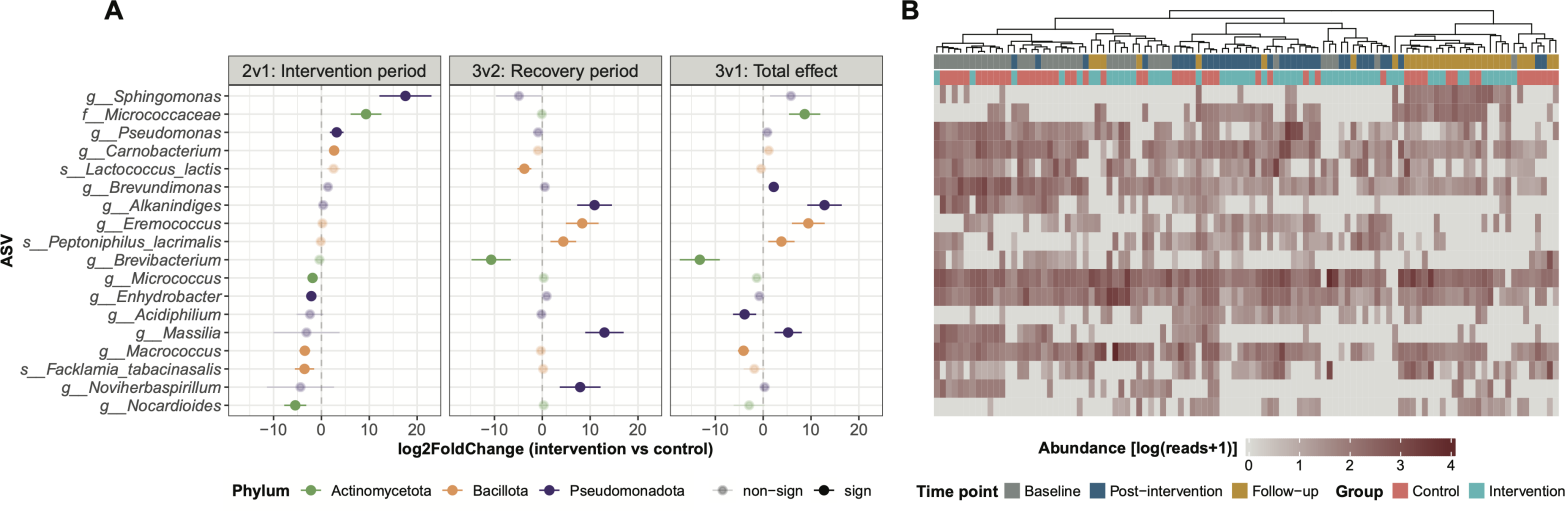
Differentially abundant taxa on hands for group × time. (**A**) ASVs identified by DESeq2 as significantly different between intervention and control groups across three time contrasts (facets). ASVs are colored by phylum and ordered by log_2_ fold change (± SE) for the intervention period (time point 2 vs1). Labels indicate the lowest classified taxonomic rank. Significance thresholds were |log_2_FC| ≥ 1 and FDR-adjusted *P* < 0.05. ASVs meeting both criteria are shown as opaque points (labeled “sign” in the inset legend), whereas semi-transparent points (labeled “non-sign”) do not meet these thresholds. (**B**) Heatmap of log₁₀(+1)-transformed abundances of differentially abundant ASVs (rows ordered as in panel A) across all hand samples (columns). Samples were hierarchically clustered by Bray-Curtis distances using Ward.D2 linkage. Annotations indicate group (pink = control, blue = intervention) and time point (gray = baseline, blue = post-intervention, gold = follow-up). Color scale ranges from low (gray) to high (red) abundance.

Next, we investigated whether particular bacteria were subject to antiseptic intervention-driven abundance shifts. At the chosen threshold (FDR *P* < 0.05, |log_2_FC| ≥ 1), 18 of 704 evaluated ASVs on hands were significantly enriched or depleted in the intervention group relative to controls in at least one contrast (Fig. 4A). Specifically, we identified nine, seven, and nine ASVs as differentially abundant (DA) between groups for the immediate intervention effect, recovery phase, and the overall study period, respectively, reflecting intervention-driven changes captured by the interaction term. DA ASVs were distributed across three phyla: *Actinomycetota*, *Bacillota*, and *Pseudomonadota*, and all were assigned to distinct genera (Fig. 4A). Seven ASVs appeared as DA in two contrasts; none of which overlapped for the intervention and recovery period. Clustering of all DA ASVs showed that samples clustered primarily by round, with groups more interspersed (Fig. 4B), consistent with the PERMANOVA results.

In comparison, in the untreated forearms, there was only one DA ASV for the interaction term out of 312 tested, namely a *Cutibacterium*, which was suppressed in the intervention group during recovery (log_2_FC = −7.68, *P* < 0.001) and overall (log_2_FC = −10.4, *P* < 0.001), and was not found to be DA in hands. Complete DESeq2 results are provided in Table S1.

## DISCUSSION

Soldiers experience substantial environmental exposure during field exercises, which can influence the bacterial communities on their skin (46). In this study, we found that such environmental factors explained more of the observed microbiome variation than which antiseptic type they used. Whereas repeated HOCl-based hand disinfection had minimal immediate effects on community diversity or composition, it was associated with a notable post-treatment increase in alpha diversity compared to standard alcohol-based hand sanitation. No group differences were seen at untreated forearm sites.

Repeated HOCl-based hand disinfection during the 10-day military field exercise had limited immediate effects on the microbiota. Alpha diversity on the hands did not change within or between groups, whereas forearm diversity increased similarly in both groups, likely reflecting shared environmental exposure during field training (46, 61). This suggests that hand disinfection in both groups suppressed the natural accumulation of environmental microbes, counteracting the increase observed on untreated skin. Vindenes et al. recently showed that alpha diversity increases with time since last handwash (19), supporting this interpretation. Several studies have also reported minimal short-term effects on microbial diversity by normal use of alcohol-based hand sanitizers, indicating some degree of resistance (62, 63). Although we detected differences in beta diversity trajectories during the exercise, these explained only a small proportion of the total variation. The HOCl-based intervention appeared to preserve greater inter-individual heterogeneity, not showing the homogenization effect previously demonstrated in the control group (46), and similar patterns of higher between-participant variation among those not using alcohol-based hand rubs have been reported elsewhere (63). However, baseline differences between groups, likely attributable to troop affiliation, limit interpretation of these compositional shifts. Differential abundance analyses identified few consistently altered taxa, with no evident phylogenetic pattern, consistent with the broad-spectrum, non-targeted nature of antiseptics (64) and supporting the conclusion that treatment effects were modest relative to environmental variation.

In contrast to the limited effects during the exercise, the post-treatment divergence suggests a formulation-specific recovery pattern. One interpretation is that the HOCl-based antiseptic, lacking the lipid-stripping properties of alcohol (65, 66), may preserve a skin environment more conducive to recolonization once both environmental and antiseptic pressures are removed (67–70). Because beta diversity trajectories during recovery were similar between groups, the rebound in the HOCl group appears to be driven primarily by increased evenness rather than directional shifts in particular taxa. Although this study cannot directly assess pathogenicity or determine whether higher diversity is beneficial, prior research links lower hand microbial diversity to increased presence of potential pathogens (63). Several studies agree that skin microbiome perturbations are generally reversible (5, 10, 18, 71); however, most have examined single applications, which do not reflect the frequent use advised in field or healthcare settings. To our knowledge, only one prior study has investigated microbiome responses to a hypochlorous acid-based hygiene solution, reporting no change in bacterial diversity after a single application to ocular skin (72). Our findings extend this literature by suggesting that formulation-specific effects may be most evident after discontinuation rather than during active use. By follow-up, both groups show similar, elevated within-group dispersion, reflecting a reassembly phase where host factors dominate. As the soldiers were on military leave during the three-week recovery period and thus no longer belonged to any group/troop, the loss of group signature and convergence toward high individualized variability was expected.

A key strength of this study is its interaction design, which enabled us to assess differences in microbiome trajectories between groups over time. Combined with longitudinal sampling at three time points, this design captured both immediate and sustained microbiome responses to repeated antiseptic exposure—dynamics often missed in studies limited to single-dose or short-term assessments (5, 10, 12, 18). We also included an untreated control site (forearms) for each participant, which is rare in similar studies (10, 65). While the statistical power of this control site was limited by low sample size, it served as an internal reference, strengthening the inference that differences at treated sites were related to the antiseptic intervention.

This study also has important limitations. Full randomization was not feasible due to logistical constraints, resulting in baseline differences in beta diversity between groups, limiting inference. The interaction-based models partially mitigated, but did not eliminate, troop as a potential confounder by comparing within-individual trajectories rather than static between-group differences. In addition, we did not measure direct antibacterial efficacy or viable load, and differences in bactericidal activity between formulations could remain undetected since DNA-based profiling does not differentiate between live and dead cells. Furthermore, antiseptic usage was relatively low and variable, but self-reported application frequencies matched measured quantities and were similar across both groups. While the dose-response analyses should be interpreted with caution, as it is an indirect and uncertain estimate of actual skin application, they do suggest that dosage had minimal impact on the primary outcomes. Finally, while not all contributing factors could be controlled, especially during the recovery period when the soldiers were on leave, there is strength in evaluating the intervention under operationally relevant conditions.

Overall, our findings indicate that environmental exposure dominates microbiome shifts during field exercises, whereas antiseptic formulation predominantly influences recovery dynamics. The HOCl-based formulation was associated with a significant increase in bacterial alpha diversity after discontinuation. While the clinical implications of increased diversity cannot be fully inferred here, this pattern, together with reported wound healing properties and reduced subjective discomfort associated with HOCl (29, 32, 34, 39, 43), supports its potential suitability in military settings. Studies that directly compare antimicrobial activity against common pathogens alongside microbiome and skin-tolerance outcomes are warranted to clarify its role as an alternative antiseptic. Future research should also investigate the effects of prolonged antiseptic use, tracking soldiers over extended periods to correlate microbiome changes and antiseptics with reported illness, leveraging their monitored and uniform lifestyle.

## Supplementary Material

Reviewer comments

## Data Availability

The sequencing data have been deposited in the NCBI Short Read Archive under BioProject accession PRJNA1159222. Code used to analyze the data can be found at: https://github.com/Rounge-lab/skin_microbiome_16S_seq_analyses.

## SUPPLEMENTAL MATERIAL

Supplemental figures spectrum.03771-25-s0001.pdf Fig. S1 to S5.

Table S1 spectrum.03771-25-s0002.xlsx Differential abundance.

OPEN PEER REVIEW reviewer-comments.pdf An accounting of the reviewer comments and feedback.

ASM does not own the copyrights to Supplemental Material that may be linked to, or accessed through, an article. The authors have granted ASM a non-exclusive, world-wide license to publish the Supplemental Material files. Please contact the corresponding author directly for reuse.
