# Supplementary material for: Longitudinal analysis of the hand microbiome in response to chlorine-based antiseptic use during a military field exercise
Source: Microbiol Spectr. 2026 Jul 14;14(8):e03771-25. doi: 10.1128/spectrum.03771-25 (PMC13436202; doi:10.1128/spectrum.03771-25)
Supplement: Supplementary file 1 [file reviewer-comments.pdf]

AMERICAN SOCIETY FOR MICROBIOLOGY | Microbiology Spectrum

Longitudinal analysis of the hand microbiome in response to chlorine-based antiseptic use during a military field exercise

Susanne Glenna, Einar Birkeland, Russell Orr, Gregor Gilfillan, Marianne Dalland, Ole Andreas Økstad, Øyvind Voie, and Trine Rounge

Corresponding Author(s): Trine Rounge, Universitetet i Oslo

Review Timeline:

Submission Date:	November 21, 2025
Editorial Decision:	March 10, 2026
Revision Received:	May 8, 2026
Accepted:	June 2, 2026

Editor: Xinzhao Tong

Reviewer(s): Disclosure of reviewer identity is with reference to reviewer comments included in decision letter(s). The following individuals involved in review of your submission have agreed to reveal their identity: Zhu Xiaoyu (Reviewer #3)

Transaction Report:

(Note: With the exception of the correction of typographical or spelling errors that could be a source of ambiguity, letters and reports are not edited. The original formatting of letters and referee reports may not be reflected in this compilation.)

DOI: https://doi.org/10.1128/spectrum.03771-25

Re: Spectrum03771-25 (**Longitudinal analysis of the hand microbiome in response to chlorine-based antiseptic use during a military field exercise**)

Dear Prof. Trine B Rounge:

Thank you for the privilege of reviewing your work. Below you will find my comments, instructions from the Spectrum editorial office, and the reviewer comments.

The reviewers found your study and the data you have collected to be valuable. However, a key concern was raised regarding the interpretation of your results. I agree with the reviewer that while the phenomena you observed are interesting, the conclusions drawn in the current version of the manuscript need to be more carefully aligned with the study's design.

Please return the manuscript within 60 days; if you cannot complete the modification within this time period, please contact me. If you do not wish to modify the manuscript and prefer to submit it to another journal, notify me immediately so that the manuscript may be formally withdrawn from consideration by Spectrum.

Revision Guidelines
To submit your modified manuscript, log into the submission site at https://spectrum.msubmit.net/cgi-bin/main.plex. Go to Author Tasks and click the appropriate manuscript title to begin. The information you entered when you first submitted the paper will be displayed; update this as necessary. Note the following requirements:

• Upload point-by-point responses to the issues raised by the reviewers in a file named "Response to Reviewers," NOT in your cover letter.
• Upload a compare copy of the manuscript (without figures) as a "Marked-Up Manuscript" file.
• Upload a clean .DOC/.DOCX version of the revised manuscript and remove the previous version.
• Each figure must be uploaded as a separate, editable, high-resolution file (TIFF or EPS preferred), and any multipanel figures must be assembled into one file.
• Any supplemental material intended for posting by ASM should be uploaded with their legends separate from the main manuscript. You can combine all supplemental material into one file (preferred) or split it into a maximum of 10 files with all associated legends included.

For complete guidelines on revision requirements, see our Submission and Review Process webpage. Submission of a paper that does not conform to guidelines may delay acceptance of your manuscript.

Data availability: ASM policy requires that data be available to the public upon online posting of the article, so please verify all links to sequence records, if present, and make sure that each number retrieves the full record of the data. If a new accession number is not linked or a link is broken, provide Spectrum production staff with the correct URL for the record. If the accession numbers for new data are not publicly accessible before the expected online posting of the article, publication may be delayed; please contact production staff (Spectrum@asmusa.org) immediately with the expected release date.

Publication Fees: For information on publication fees and which article types are subject to charges, visit ourwebsite. If your manuscript is accepted for publication and any fees apply, you will be contacted separately about payment during the production process; please follow the instructions in that e-mail. Arrangements for payment must be made before your article is published.

ASM Membership: Corresponding authors may join or renew ASM membership to obtain discounts on publication fees. Need to upgrade your membership level? Please contact Customer Service at Service@asmusa.org.

The ASM Journals program strives for constant improvement in our submission and publication process. Please tell us how we can improve your experience by taking this quick Author Survey.

Thank you for submitting your paper to Spectrum.

Sincerely,
Xinzhao Tong
Editor
Microbiology Spectrum

Reviewer #1 (Comments for the Author):

While the manuscript does not explicitly measure functional parameters of skin barrier health, it provides a technically robust and scientifically rigorous longitudinal dataset regarding the "recovery phase" of the skin microbiome-a stage that remains

significantly underexplored in clinical literature. The study effectively addresses a defined operational need in military medicine by validating a multipurpose, non-alcoholic antiseptic that is safe for both intact and compromised skin, offering a sustainable alternative to standard alcohol-based protocols. Given that the authors' previous baseline ecological work was published in Microbiology Spectrum in April 2025, this subsequent intervention study acts as a natural "Resource Report" extension. I am also intrigued by the discovery of a significant group x time interaction for alpha diversity and the identification of 18 treatment-specific differentially abundant ASVs.

Reviewer #3 (Comments for the Author):

1. This study possesses a degree of novelty. It represents the first longitudinal evaluation of the effects of stabilized hypochlorous acid (HOCl) antiseptic on the skin microbiome conducted under real-world field conditions. As the authors note in the Discussion (p. 23), the only prior related study examined the effects after a single application (Reference 72). Embedding the study within a NATO military exercise provides strong ecological validity, offering a more realistic reflection of actual usage scenarios compared to laboratory-controlled settings. The inclusion of a recovery time point (3 weeks post-cessation) is a notable strength of the study design. Most comparable studies focus exclusively on immediate effects, whereas the present study found that differential effects between antiseptics were most pronounced after cessation rather than during the intervention period - a finding that is conceptually novel and underscores the value of longitudinal designs. Additionally, the use of the forearm as an within-subject untreated control enhances the credibility of causal inference.
2. The overarching research framework of comparing the effects of different antiseptics on the skin microbiome is not entirely new (References 10, 12, and 18 provide precedent). The methodological tools employed (16S rRNA amplicon sequencing, PERMANOVA, DESeq2, etc.) are all standard approaches in the field, with no methodological innovation introduced. Furthermore, the sample size is relatively small (n = 39), and the HOCl product tested is a commercial product (SafeDes{trade mark, serif}), with the study supported through an industrial PhD program funded by the manufacturer. Although the authors declare no conflict of interest, the relationship between the funding source and the directionality of the study conclusions warrants attention.
3. For a study that aims to recommend an alternative antiseptic product for military personnel, the complete absence of any evaluation of the actual bactericidal or bacteriostatic efficacy of either antiseptic (e.g., clearance rates of common pathogens) leaves the conclusion of HOCl as a "promising alternative" without critical supporting evidence. The authors should, at minimum, explicitly acknowledge this gap in the Discussion and temper the tone of product endorsement.
4. Participants were assigned to groups based on military unit affiliation rather than random allocation, resulting in significant baseline differences in beta diversity. Although the authors acknowledge this limitation, they do not adequately discuss how it may affect all downstream analyses. The authors are advised to conduct sensitivity analyses - for example, repeating the primary analyses on a subset of participants with similar baseline compositions, or employing propensity score matching methods.
5. Participant behavior during the three-week military leave was entirely uncontrolled, which substantially undermines causal attribution of recovery-phase differences to antiseptic type. The Discussion should more rigorously address this limitation and reduce the certainty of the corresponding conclusions.
6. The Abstract, IMPORTANCE section, and Discussion employ causal or quasi-causal language in multiple instances (e.g., "permits," "supporting," "promotes"). These should be uniformly replaced with associative language (e.g., "associated with," "correlated with"). In particular, the conclusion that HOCl represents a "promising alternative" lacks adequate support in the absence of comparative antimicrobial efficacy data.
7. Table 1 shows that at baseline, only 8 forearm samples from the control group and 5 from the intervention group passed quality control - far fewer than the hand samples. This severely limits the statistical power of the forearm analyses, which serve as a critical internal control. The authors should explain in the Methods section why forearm sample attrition was so extensive, and discuss in the Results section how this affects the interpretation of the "no difference" finding for forearm samples - the null result could represent a true negative or equally a false negative due to insufficient power.
8. 16S amplicon sequencing cannot distinguish between DNA from viable and non-viable bacteria. This is particularly relevant in antiseptic research, as antiseptics may kill bacteria while their DNA remains detectable. This methodological limitation should be discussed.
9. Nine participants in the intervention group owned pets compared to only two in the control group (Table 1), with seven owning dogs. Pets, particularly canines, have been shown to significantly influence the human skin microbiome. Although the authors tested the correlation between pet ownership and recovery-phase diversity changes ($r = 0.096$, $P = 0.72$), a Pearson correlation alone may be insufficient to rule out this confounding factor. It is recommended that pet ownership be included as a covariate in the mixed-effects models.
10. The volume of antiseptic used in the intervention group varied considerably (14-128 mL), with estimated daily applications ranging from 1 to 5 times. Although the dose-response analysis did not reveal a significant association, such wide variability in usage may obscure true effects. Stratified subgroup analyses by usage volume are recommended.
11. The inclusion of counterfactual dashed lines in Figure 2 is commendable and aids in the interpretation of treatment effects. However, in Figure 4A, multiple ASVs display very large log2FC error bars, some of which cross zero. The figure legend should more clearly indicate which ASVs are statistically significant versus non-significant.
12. The Discussion section (pp. 22-25) is overly lengthy, with several arguments repeated across paragraphs. It is recommended that the Discussion be streamlined, with core arguments consolidated into three to four paragraphs, avoiding repeated emphasis on "HOCl as a promising alternative." The final paragraph of the Introduction, which discusses the

relationship to a previously published paper (Reference 46), should also be made more concise, as it currently reads with considerable overlap.

The central issue with this manuscript is that the conclusions exceed what the data can substantiate. This manifests on several levels.

First, the authors employ causal language throughout the Abstract and Discussion ("permits faster recovery," "supports recolonization"), yet the study design is a non-randomized observational study, and the three-week recovery period was entirely uncontrolled. At most, the data can demonstrate association, not causation.

Second, the authors' central claim - that HOCl is more conducive to microbiome recovery than alcohol-based antiseptics - lacks a critical prerequisite: whether the antimicrobial efficacy of the two products is comparable. If HOCl is inherently less bactericidal than alcohol, then the higher diversity observed during recovery may simply reflect less killing rather than a gentler preservation of the microbiome. Without a comparative efficacy assessment, the conclusion that HOCl represents a "promising alternative" is not well-founded.

Third, beta diversity already differed significantly between the two groups at baseline, meaning that all subsequent between-group differences could represent a continuation of pre-existing differences rather than a true intervention effect. Although the authors acknowledge this point, they do not sufficiently temper their conclusions accordingly.

In summary, the data themselves are valuable and the observed phenomena are real, but there is a logical leap between what was observed and the conclusions the authors draw - one that lacks a sufficient chain of evidence to bridge.

Point-by-point response, Glenna et al, "Longitudinal analysis of the hand microbiome in response to chlorine-based antiseptic use during a military field exercise"

Response to Reviewer #1,

Thank you for the very kind words and appraisal of our manuscript. This is indeed an extension of our previous work published in *Microbiology Spectrum*, and we hope it will be a valuable contribution to the journal.

Response to Reviewer #3,

Thank you very much for your thorough review and valuable feedback on our paper. We appreciate your concerns and kindly refer you to the marked-up manuscript for review of adjustments we have made based on your feedback. Line numbers in our responses refer to numbers in the final revised manuscript.

- **Concern #1 (point 2)**: Potential conflict of interest

  Response: Thank you for pointing out the potential conflict of interest, and we agree that we should address this in the paper. The project was funded by the Norwegian Research Council's Industrial PhD programme, which involved self-financing from SoftOx Solutions AS, the company where the Industrial PhD was based. There is no commercial use of the results beyond SoftOx Solutions being able to reference the published work in the marketing of their products. None of the researchers involved in the project hold shares or ownership interests in the company. The first author of this manuscript is a PhD candidate who has had an employment relationship with the company (from aug 2021-oct 2024), but this was only a requirement in order to receive support from the Industrial PhD programme. The candidate has since the start of the project been a visiting researcher at Norwegian Defense Research Establishment (Forsvarets forskningsinstitutt, FFI), and has since the end of 2024 (when the

industrial PhD program was officially terminated), been fully employed by FFI. All scientific work with this study has been led by FFI, and none of the authors have any conflicts of interest. We have added a “Conflict of interest” statement in the revised manuscript (lines 437-443).

- **Concern #2 (point 3 and 13b)**: Missing bactericidal efficacy (and comparison)

  Response: We thank the reviewer for this important comment. The scope of this paper was not to compare the antibacterial efficacy of two antiseptic products, but we agree that we should avoid a tone of product endorsement. We acknowledge that, for infection-control decision-making, a “promising alternative” product is generally expected to combine a favorable microbiome/skin-tolerance profile with at least comparable antimicrobial efficacy and safety. Comparative efficacy testing was outside the scope of this microbiome-focused study, but the reviewer is right to highlight that we cannot from our data alone claim that stabilized HOCl can *replace* alcohol-based products for infection control without pathogen clearance data. In the present study we relied on existing evidence that the HOCl + acetic acid formulation is EN 1500/13727/13624-approved and has documented broad-spectrum antimicrobial and antibiofilm activity in other settings (1-3), but we did not perform a head-to-head comparison to alcohol-based products and cannot ensure that viable load or killing efficacy is equivalent. However, we do not think that an efficacy assessment is strictly necessary to support our main claim that “in this cohort, HOCl use was associated with higher post-treatment bacterial DNA diversity on hands compared with standard alcohol-based practice.” Furthermore, our results weaken the “purely less-killing” explanation since there was no difference between HOCl group vs controls immediately post-intervention. In response to the concern, we have revised the Discussion (lines 403-405) to explicitly acknowledge that we did not assess comparative antimicrobial efficacy and that this gap limits the inference of product utility. We add a caveat that stabilized HOCl may represent a promising candidate for further evaluation as an alternative to alcohol-based antiseptics, contingent on future studies that directly compare antimicrobial performance (including common pathogens) alongside microbiome and skin-tolerance outcomes.

- **Concern #3 (point 4 and 13c)**: Groups/troops different at baseline

  Response: We acknowledge that allocating participants by military unit rather than through randomization is a limitation. This design was necessary because individual randomization was not feasible in this operational field setting. Such quasi-experimental designs estimate intervention effects using pre-existing or non-equivalent groups when randomization is impractical or unethical (4). However, the resulting baseline difference in hand beta diversity complicates interpretation of subsequent between-group contrasts and limits causal inference. To mitigate this confounder, we used appropriate statistical methods, including difference-in-differences (DiD) modeling when possible. These interaction-based models are designed to account for stable pre-existing differences and to test whether *within-individual trajectories* diverge after the intervention rather than whether static group means differ. While this approach cannot eliminate confounding from baseline structure, it reduces its influence and shifts the focus to changes over time. The lack of group × time effects at the untreated forearm site supports the interpretation that hand-specific effects are intervention-related rather than extensions of baseline differences, although we acknowledge the limited power at this site (concern #6).

  We considered the suggested sensitivity analyses, but our sample size and the high dimensionality of microbiome data make matching-based approaches statistically unstable and underpowered, as they would result in substantial data loss without ensuring improved confounding control. Accordingly, we have revised the Discussion (lines 399-403) to more clearly state that baseline differences constrain causal interpretation and that our results should be viewed as trajectory-level associations consistent with an antiseptic-related effect, not definitive causal effects.

- **Concern #4 (point 5)**: 3W recovery period uncontrolled

  Response: We agree that the three-week recovery period occurred under uncontrolled, free-living conditions and that this limits causal interpretation of recovery-phase differences. This reflects the real life of a soldier, and fully controlled experiments are only possible using animal models - with their limitations. However, we have

explicitly added the "uncontrolled" time as a limitation in the Discussion (lines 409-412). At the same time, we emphasize that the soldiers were no longer organized by their original troop assignments during the recovery phase; thus, any unmeasured behaviors during leave are unlikely to remain systematically clustered by group, and are more random with respect to the initial antiseptic allocation. This does not remove the possibility of residual confounding at follow-up, but it makes systematic, group-specific behavioral confounding less likely. Although our design cannot eliminate confounding, our longitudinal difference-in-differences framework that explores *within-individual changes* in diversity mitigates some bias and strengthens our interpreted associations.

- **Concern #5 (point 6 and 13a)**: Causative language

  Response: It was not the authors' intent to infer causation in this study, but we acknowledge that the original wording to some extent implied causality. We agree that given the quasi-experimental study design, causal language should be used very cautiously, and even though the authors believe caution has already been taken using words like "suggest", "indicate", "may", we have made several changes throughout the revised manuscript as requested. We have also significantly reduced the emphasis on product endorsement.

- **Concern #6 (point 7)**: Loss of forearm samples (low power)

  Response: Skin samples are known to have a low microbial biomass, which makes them challenging to work with. Loss of forearm samples was mainly due to insufficient sequencing depth resulting from the very low DNA amount. As demonstrated in our earlier work (Glenna et al, 2025), Supplementary Figure S1 shows that all measured forearm samples contained lower DNA concentrations than hand samples. For convenience, we reproduce this figure below.

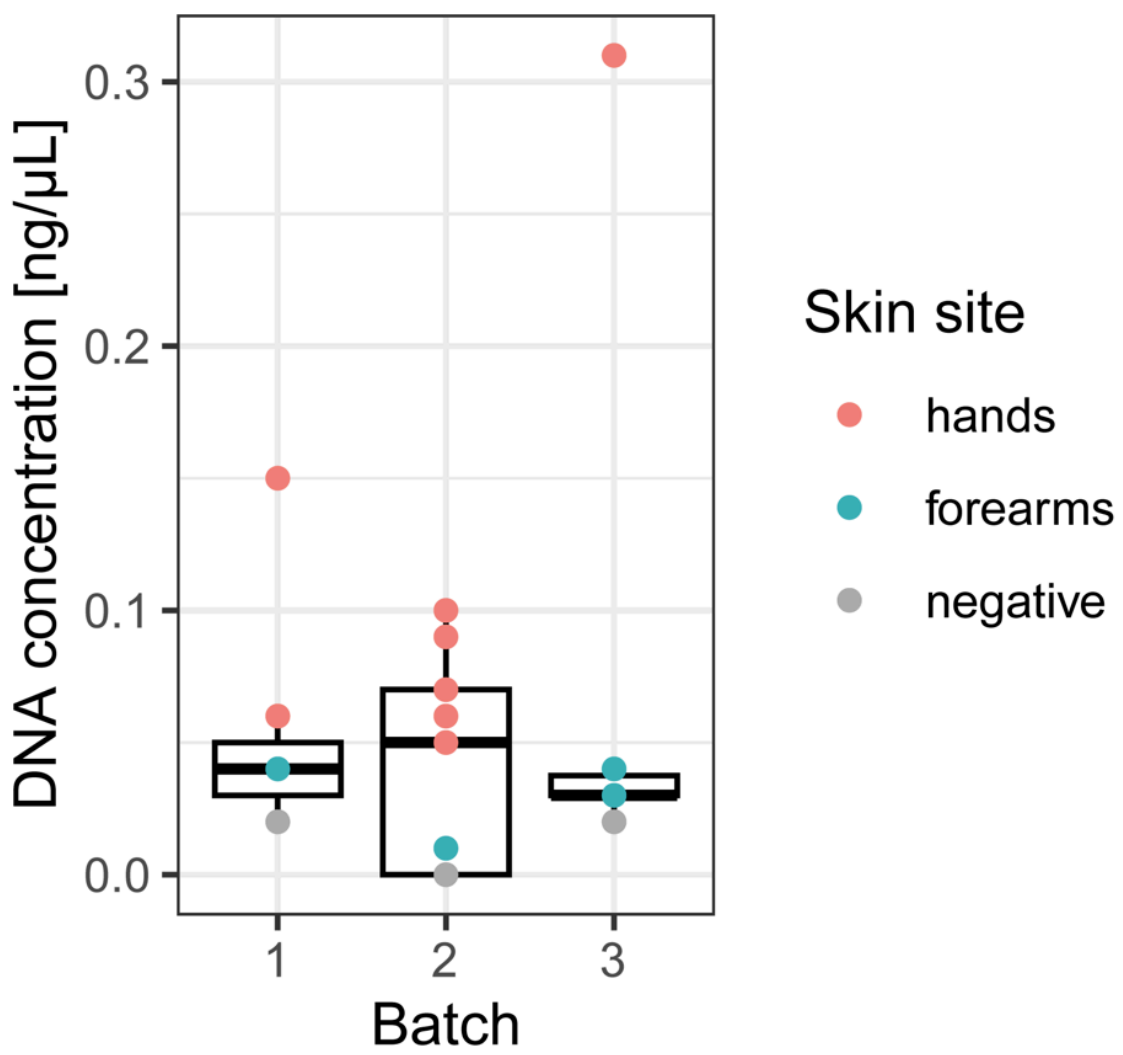

Figure S1 (reproduced from *Glenna et al., 2025, doi: https://doi.org/10.1128/spectrum.02953-24).* Provided for reviewer reference only. The figure shows the total DNA concentration [ng/µl] in subset samples (n=22) taken from hands, forearms and negative controls (air swabs) per extraction batch.

The extensive attrition of forearm samples during pre-processing was driven by objective read-quality criteria applied uniformly across all sample types. During trimming of raw reads, we decided on a threshold of 5000 trimmed reads to ensure adequate data quality and reliable representation of microbial communities in each sample. This threshold resulted in removal of 10/106 forearm samples, including most negative controls. After dada2-filtering, we chose to normalize all samples to 7000 high-quality reads based on rarefaction curves, keeping most of the diversity of the data. This step excluded another 38 forearm samples, but these would have been lost even with rarefying to 5000 reads (we chose the highest sample depth that would keep the same amount of samples overall). We agree that this attrition limits the statistical power of the forearm analyses, and have been more transparent about it in the revised manuscript (lines 228-230 and 395-398, including a clarified methods section (lines 151-165). Further, the sequencing data results have been updated, with numbers for the 163 samples that were included in downstream analysis (lines 226-227).

- **Concern #7 (point 8)**: 16S limitation (live vs dead)

  Response: 16S rRNA amplicon sequencing, as applied here, is a widely used, standard method in microbiome research that inherently measures total bacterial DNA without

distinguishing viable from non-viable cells. Because this limitation is common to essentially all 16S studies and our sequencing workflow followed established protocols, we did not originally highlight it in the Discussion. We agree, however, that in the context of antiseptic research this issue is particularly relevant, since the detectable signal from residual DNA of recently killed cells may overestimate the persistence of taxa that are effectively inactivated by the antiseptics. We have revised our manuscript to include this methodological limitation (see lines 403-405).

- **Concern #8 (point 9)**: Pet ownership

  Response: As the reviewer points out, pet ownership is unevenly distributed between groups and pets can influence the skin microbiome. As reported in the Results, we already examined whether the observed increase in hand alpha diversity during recovery in the intervention group was correlated with pet ownership and found no association (Pearson's r = 0.096, *P* = 0.72). We acknowledge that this bivariate correlation does not fully exploit the longitudinal design, and we agree that a model-based adjustment is more robust. In response, we have therefore performed a sensitivity analysis in which pet ownership was added as a binary covariate to our linear mixed-effects model for hand alpha diversity (with Shannon diversity as the example), using the same framework described in Methods. A likelihood ratio test comparing the original model and the pet-adjusted model showed that inclusion of pet ownership as a fixed effect did not significantly improve model fit (ΔAIC = +2, L.ratio = 0.026, $p = 0.87$). Moreover, the overall group × time interaction remained significant in the adjusted model (F = 4.058, $p = 0.0222$), and the coefficient for pet ownership was negligible and non-significant (F = 0.0021, $p = 0.9636$). Difference-in-differences contrasts from emmeans were essentially unchanged from the original model; intervention phase (-0.171, $p = 0.5523$), recovery phase (+0.823, $p = 0.0084$). Since the group × time interaction remains statistically significant and stable, and the pet_owner term neither improves fit nor materially alters the estimates, we conclude that pet ownership does not drive the observed interaction effect. In the revised manuscript, we have replaced the correlation analysis with this sensitivity analysis (lines 181-183 and 252-257), showing that adjusting for pet ownership gave similar

results and that pet ownership remains, at most, a minor potential source of residual confounding.

- **Concern #9 (point 10)**: Antiseptic usage variability

  Response: There was, as the reviewer points out, substantial variation in measured antiseptic volume in the intervention group. As we agree such variability could have caused differential effects, we conducted a dose-response analysis in the setting where the strongest association was seen (i.e., the relative increase in alpha diversity in the post-intervention period). As we briefly mentioned in the discussion, this analysis should be treated with caution. The reason for caution is mainly due to our inability to accurately measure actual use patterns in the field based on self-report of application frequency and remaining antiseptic volume. Flask weight is an indirect and inherently noisy proxy for true skin exposure, as it could be influenced by factors such as spills or incomplete dispensing, and therefore does not provide a strictly controlled measure of actual application. Moreover, the study was set up to conform to the intention-to-treat principle, and any effects were considered for how the intervention was carried out in the field, rather than in an ideal setting where antiseptic use could have been strictly controlled. To clarify the limitation of the dose-response analysis, we have amended the discussion of antiseptic dosage association (lines 405-409). Although the reviewer is right in principle to point out that conducting subgroup analyses could have uncovered hidden effects, the study is already on the low end of statistical power. Combined, we believe that the uncertainty of the measure and the lack of statistical power is enough to make subgroup analyses in this setting unproductive.

- **Concern #10 (point 11)**: Figure 4A, legend not clear enough about significance

  Response: We thank the reviewer for pointing out the need for clearer indication of statistical significance in Figure 4A. The figure already distinguishes significant ASVs as opaque (‘sign’) and non-significant ASVs as semi-transparent (‘non-sign’), but this was not fully explained in the legend. We have now revised the legend (see lines 336-338) to explicitly state the significance thresholds and to link them to the point styles used in the ‘non-sign/sign’ key below the panel, so that readers can more easily

see which points meet the statistical criteria.

- **Concern #11 (point 12)**: Too long/repeated Discussion

  Response: We agree that the Discussion benefitted from being more concise. In response, we have streamlined the Discussion, focusing on reducing repetitive statements and avoiding what may be interpreted as product endorsements. We have substantially reduced the number of words from the original manuscript. At the same time, since the reviewer also requested that we add explicit discussion of several additional methodological limitations (e.g. live/dead discrimination, lack of direct efficacy comparison), the final section about limitations is still somewhat lengthy.

- **Concern #12 (point 12)**: Final part of Introduction too repetitive

  Response: We understand that by describing both the findings of our previous study and the current study design in detail, the final paragraph of the Introduction was somewhat overlapping and repetitive. To improve clarity and concision, we have now streamlined this paragraph (see lines 80-91).

References

1. Yüksel YT, Sonne M, Nørreslet LB, Gundersen G, Fazli MM, Agner T. 2022. Skin barrier response to active chlorine hand disinfectant—An experimental study comparing skin barrier response to active chlorine hand disinfectant and alcohol-based hand rub on healthy skin and eczematous skin. Skin Res Technol 28:89–97.
2. Burian, Ewa A., Lubna Sabah, Klaus Kirketerp-Møller, Elin Ibstedt, Magnus M. Fazli, og Glenn Gundersen. The Safety and Antimicrobial Properties of Stabilized Hypochlorous Acid in Acetic Acid Buffer for the Treatment of Acute Wounds - a Human Pilot Study and In Vitro Data. The International Journal of Lower Extremity Wounds 22, nr. 2 (2023): 369–77. First published online May 5, 2021. https://doi.org/10.1177/15347346211015656.
3. Aherne O, Ortiz R, Fazli MM, Davies JR. Effects of stabilized hypochlorous acid on oral biofilm bacteria. BMC Oral Health. 2022 Sep 20;22:415. doi:10.1186/s12903-022-02453-2.
4. Van Butsic, David J. Lewis, Volker C. Radeloff, Matthias Baumann, Tobias Kuemmerle, Quasi-experimental methods enable stronger inferences from observational data in ecology, Basic and Applied Ecology, Volume 19, 2017, Pages 1-10, ISSN 1439-1791, https://doi.org/10.1016/j.baae.2017.01.005.

Re: Spectrum03771-25R1 (**Longitudinal analysis of the hand microbiome in response to chlorine-based antiseptic use during a military field exercise**)

Dear Prof. Trine B Rounge:

Your manuscript has been accepted, and I am forwarding it to the ASM production staff for publication. Your paper will first be checked to make sure all elements meet the technical requirements. ASM staff will contact you if anything needs to be revised before copyediting and production can begin. Otherwise, you will be notified when your proofs are ready to be viewed.

Data Availability: ASM policy requires that data be available to the public upon online posting of the article, so please verify all links to sequence records, if present, and make sure that each number retrieves the full record of the data. If a new accession number is not linked or a link is broken, provide production staff with the correct URL for the record. If the accession numbers for new data are not publicly accessible before the expected online posting of the article, publication may be delayed; please contact ASM production staff immediately with the expected release date.

Publication Fees: For information on publication fees and which article types have charges, please visit our website. We have partnered with Copyright Clearance Center (CCC) to collect author charges. If fees apply to your paper, you will receive a message from no-reply@copyright.com with further instructions. For questions related to paying charges through RightsLink, please contact CCC at ASM_Support@copyright.com or toll free at +1-877-622-5543. CCC makes every attempt to respond to all emails within 24 hours.

ASM Membership: Corresponding authors may join or renew ASM membership to obtain discounts on publication fees. Need to upgrade your membership level? Please contact Customer Service at Service@asmusa.org.

Embargo Policy: A press release may be issued as soon as the manuscript is posted on the Spectrum Latest Articles webpage. The corresponding author will receive an email with the subject line "ASM Journals Author Services Notification" when the article is available online.

The ASM Journals program strives for constant improvement in our submission and publication process. Please tell us how we can improve your experience by taking this quick Author Survey.

Thank you for submitting your paper to Spectrum.

Sincerely,
Xinzhao Tong
Editor
Microbiology Spectrum

Reviewer #3 (Comments for the Author):

The authors have thoroughly addressed the concerns raised in the first round of review.
