# Supplementary material for: Longitudinal analysis of the hand microbiome in response to chlorine-based antiseptic use during a military field exercise
Source: Microbiol Spectr. 2026 Jul 14;14(8):e03771-25. doi: 10.1128/spectrum.03771-25 (PMC13436202; doi:10.1128/spectrum.03771-25)
Supplement: Supplementary file 2 [file spectrum.03771-25-s0001.pdf]

SUPPLEMENTAL MATERIAL

Supplementary figures

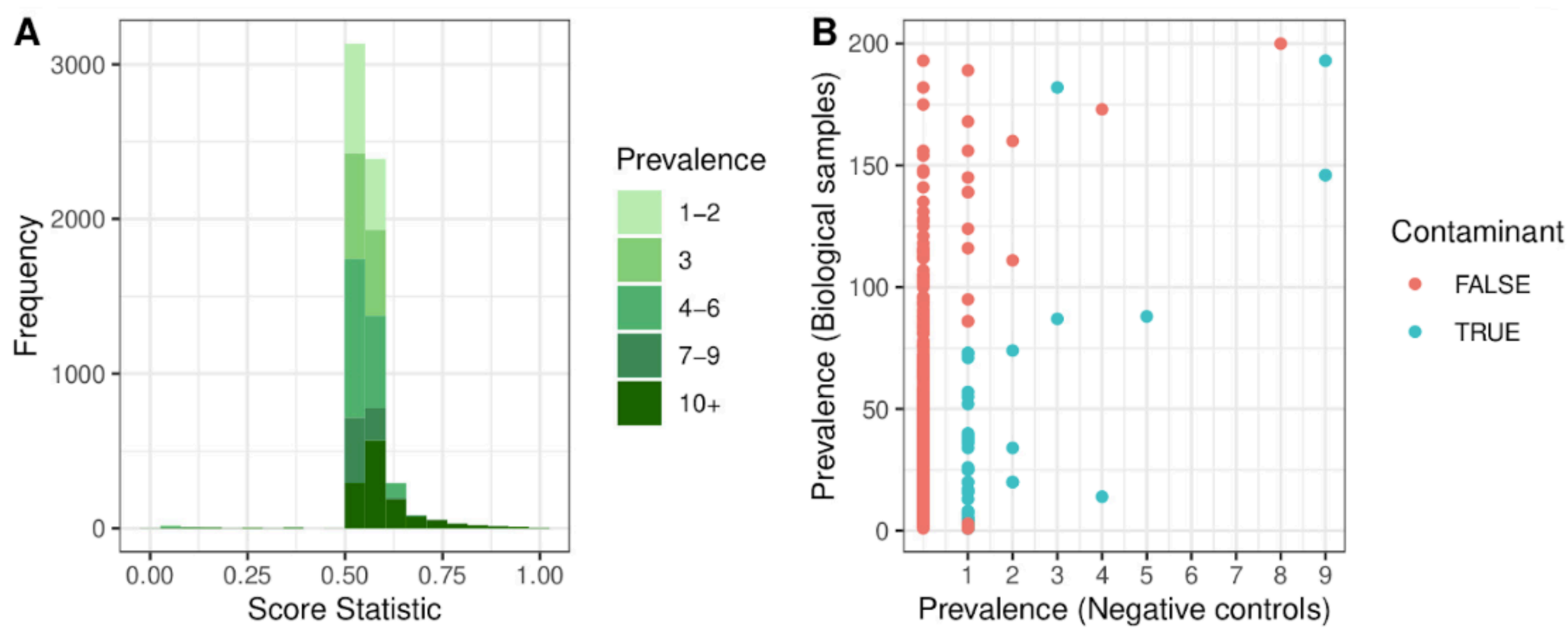

Figure S1: ***Decontam()* results. (A)** Distribution of score statistics from *decontam* assigned to each amplicon sequence variant (ASV) based on prevalence (color intensity indicating the total number of samples each ASV was present in (i.e, prevalence). A score < 0.5 indicates a contaminant. **(B)** Prevalence plot of present/absent ASVs in true samples vs negative controls. 52 contaminant ASVs identified by *decontam* (with prevalence method) are colored blue. These represented 440,796 reads, accounting for ~9% of the (non-chimeric) reads overall.

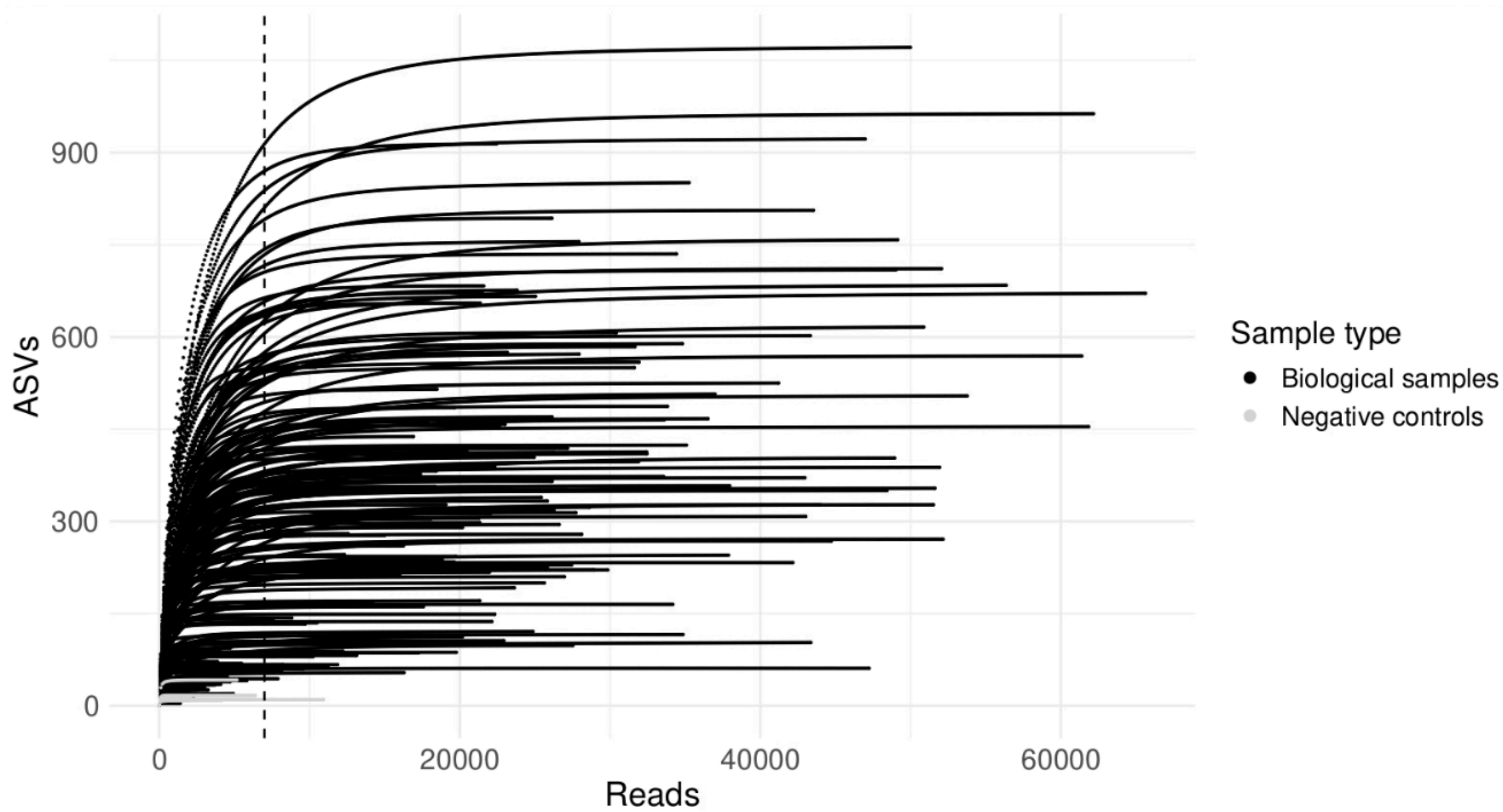

Figure S2: **Rarefaction curves.** Number of amplicon sequence variants (ASVs) per sample size (number of trimmed reads) for all biological samples (black, n=202) and negative controls (gray, n=9). The dotted line represents the chosen sampling depth of 7000 reads, which retains most of the data's diversity (where most samples have reached a plateau of new ASVs per increased depth) and the number of samples.

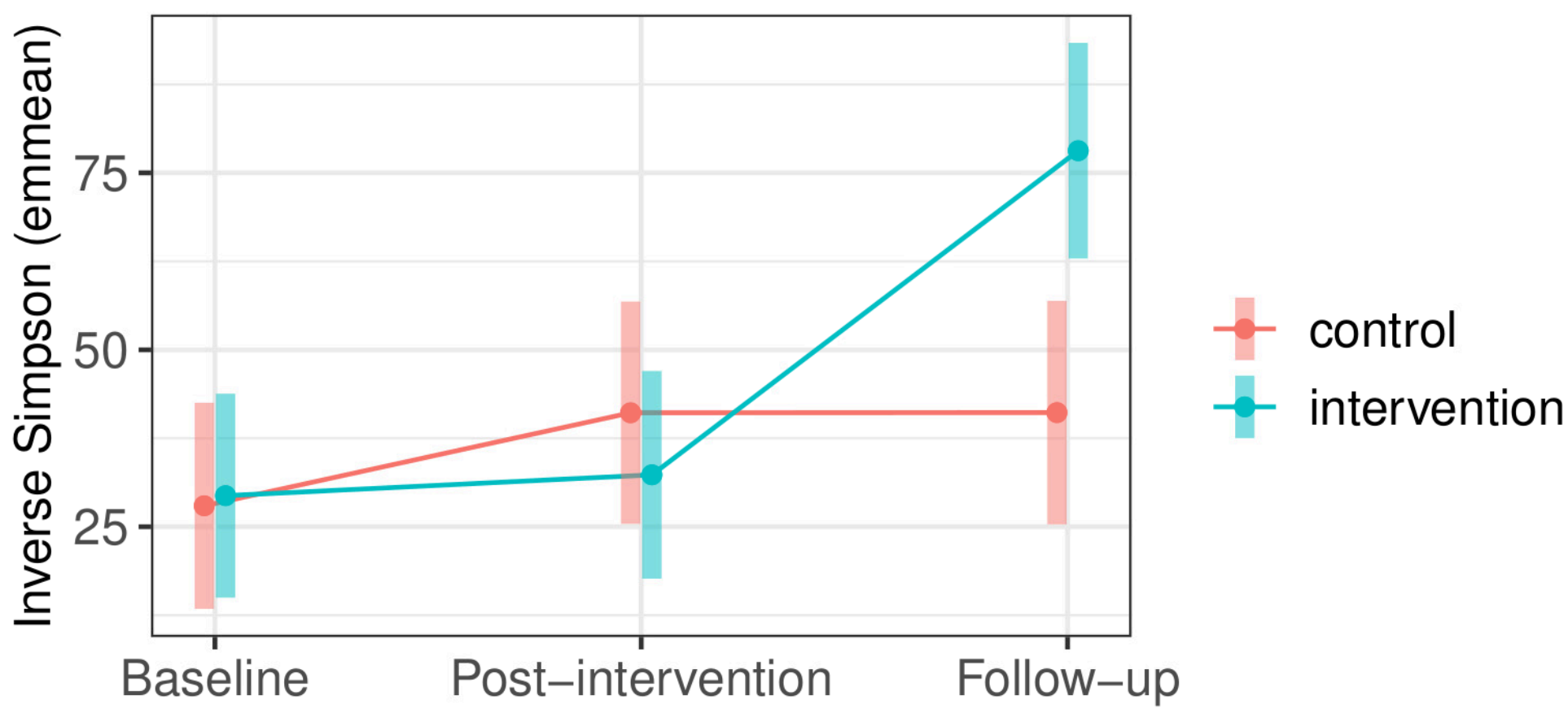

Figure S3: Inverse Simpson. Estimated marginal means of Inverse Simpson diversity for hand samples over time, comparing the two groups. This metric exhibits a similar temporal pattern to Shannon diversity (see Fig. 1A).

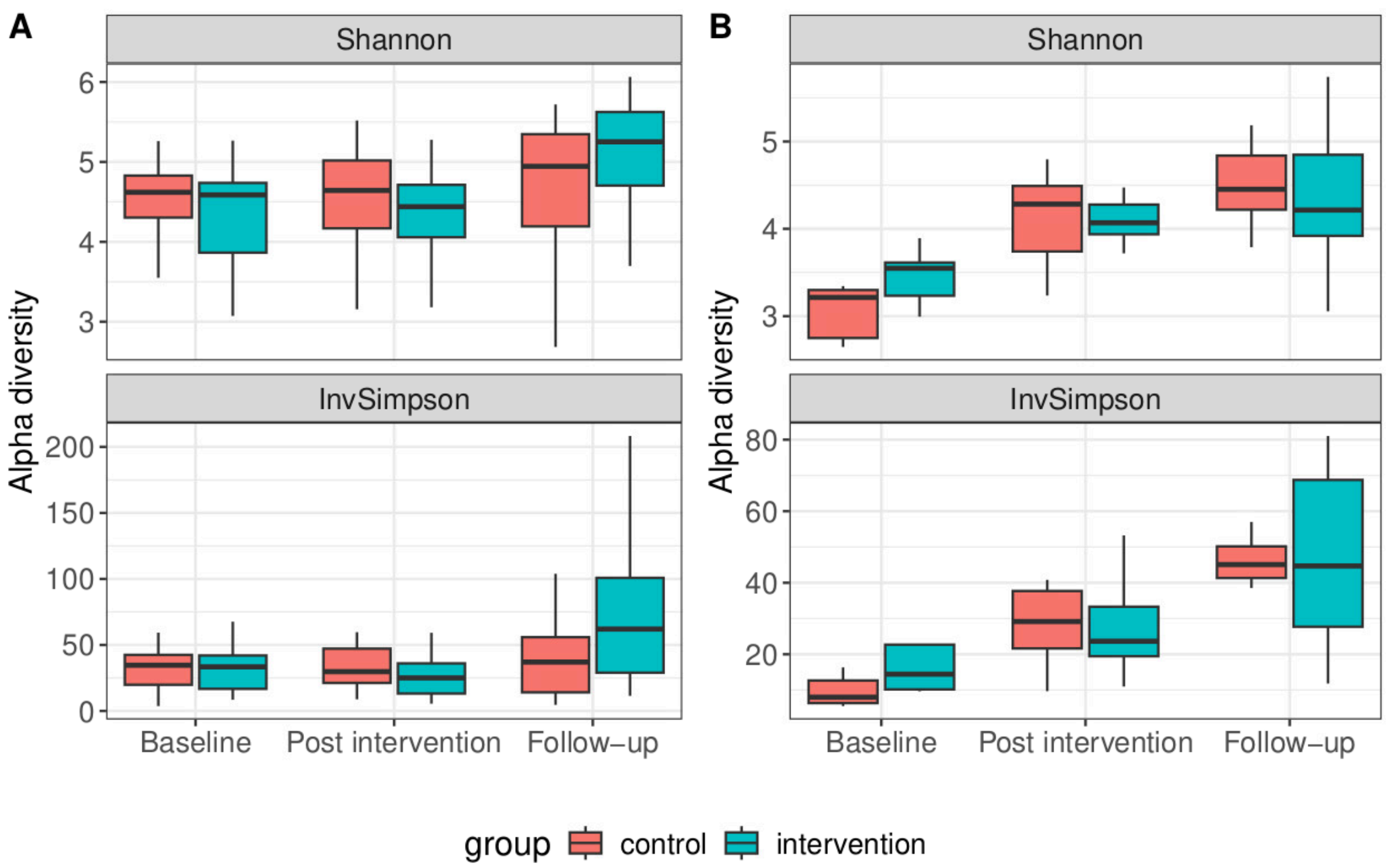

Figure S4: **Alpha diversity (unadjusted) over time.** (**A**) hands, (**B**) forearms. (Unadjusted) Shannon and Inverse Simpson diversity metrics for each group (pink = control, blue = intervention) over time points.

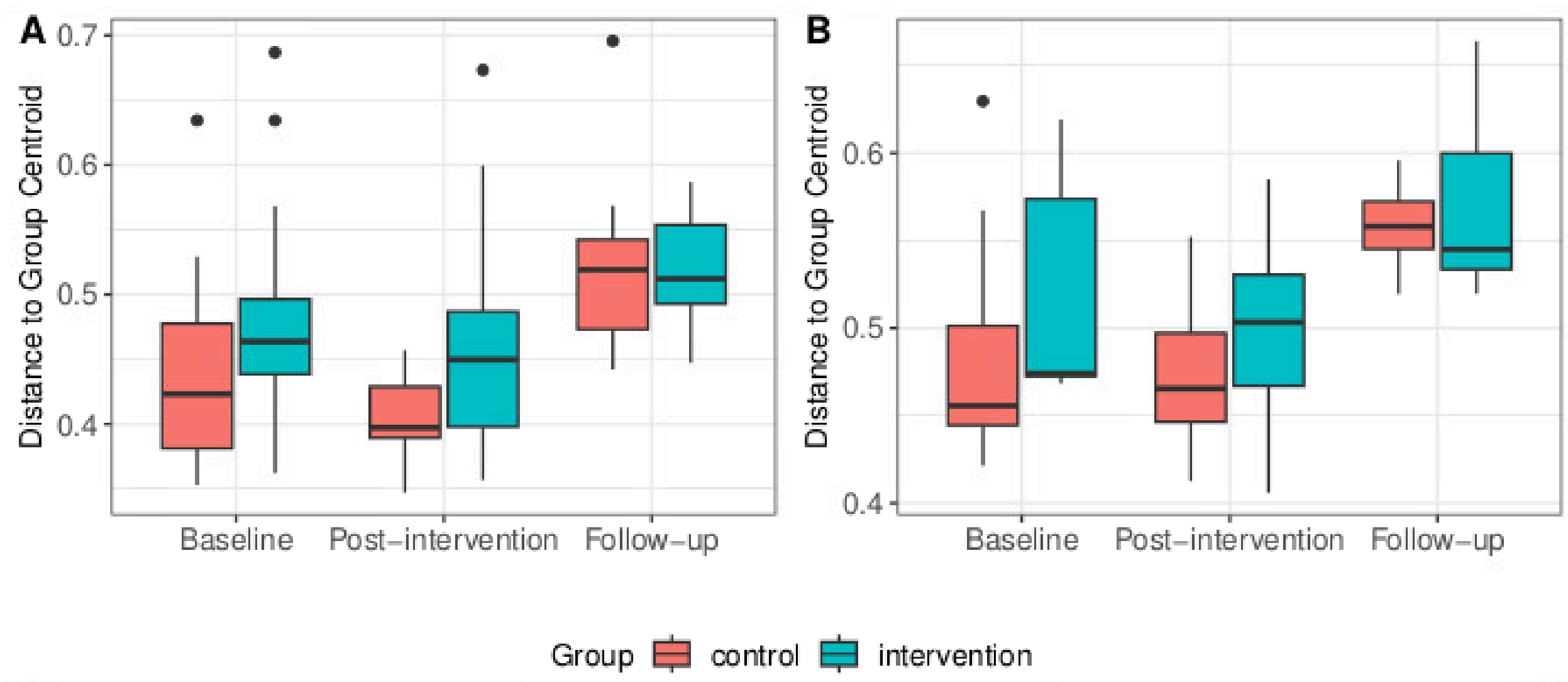

Figure S5: **Beta dispersion**. Distances to group centroids for each group (pink = control, blue = intervention) and time point in each skin site, **(A)** hands, **(B)** forearms.

Supplementary tables

Table S1: Differential abundance table (uploaded)
